# Mindfulness-Based Versus Story Reading Intervention in Public Elementary Schools: Effects on Executive Functions and Emotional Health

**DOI:** 10.3389/fpsyg.2021.576311

**Published:** 2021-07-08

**Authors:** Claudete A. R. Milaré, Elisa H. Kozasa, Shirley Lacerda, Carla Barrichello, Patricia R. Tobo, Ana Lucia D. Horta

**Affiliations:** ^1^Paulista School of Nursing, Federal University of São Paulo, São Paulo, Brazil; ^2^Hospital Israelita Albert Einstein, São Paulo, Brazil; ^3^Natura Cosméticos, Cajamar, Brazil

**Keywords:** mindfulness, children, executive function, attention, education, mental health, story reading

## Abstract

**Introduction:**

In this study we compared the effects of a mindfulness-based intervention (MBI) with a story reading intervention (SI) on the executive functions and psychological profile of children in two different public schools in São Paulo, Brazil.

**Methods:**

In this controlled clinical trial, 207 children aged 8 to 9 years old responded to the Five-Digit Test (FDT), stress levels, depression, anxiety, positive and negative affect, at baseline (T0) and 8 weeks later (T1). From T0 to T1, school 1 participated in MBI classes and school 2 in IS classes.

**Results:**

In school 1 (MBI), children improved their scores on all tests except reading (errors) and counting (errors) compared with school 2. No differences were observed between groups in terms of emotional health.

**Conclusion:**

It is feasible to implement MBI or SI in Brazilian public schools. Students in the MBI group presented broader effects in executive functions, while students in the SI group showed a trend toward reduced negative affect and depression symptoms.

## Highlights

This study contributes to the scientific evidence of the positive effects of Mindfulness and Story reading on executive functions and emotional well-being in children. Neither intervention had significant effects on depression, anxiety, stress, positive, and negative affect (although Story reading showed a trend in reducing negative affect and depression), while the Mindfulness-Based Intervention had relatively broader effects on executive functions.

## Introduction

Schools have a crucial role in the promotion of emotional health and well-being of children. In a systematic review, the authors found that greater effectiveness is achieved on emotional health when interventions focused on the promotion of self-esteem, mental health and coping outcomes of the students, rather than the prevention of mental health problems were implemented at schools (Green et al., 2005).

Social and emotional learning (SEL) programs can improve emotional health, enhancing children’s confidence in themselves and reducing conduct problems while promoting positive behaviors. In the long-term, children with greater social emotional competence are more likely to be have positive better mental health and relationships (Greenberg et al., 2017).

Positive effects on mental health and quality of life among children and adolescents have been reported when mindfulness is used as the main intervention but also when used as one resource within socioemotional development programs. Social-emotional learning (SEL) programs involving attention and care for others have yielded improvements in cognitive and physiological stress control, empathy, perspective gaining, emotional control, optimism, school self-evaluation, and attention in elementary school children (Schonert-Reichl et al., 2015). There has been a recent surge in mindfulness-based interventions developed for children, mainly in school contexts (Felver et al., 2016). These programs implement simple techniques that aim to increase self-awareness and help regulate attention, emotions and behavior in children and adolescents (Britton et al., 2014), with some reporting positive effects on school performance, psychological well-being, self-esteem and social skills (Rempel, 2012). In the Mindfulness literature, several of the functions targeted in these interventions are referred to as executive functions (EF).

In their model, Miyake et al. (2000) present three components they deem essential for executive functioning: updating (allows monitoring, manipulating and updating information from working memory), inhibition (capacity to controllably inhibit automatic responses when required) and shifting (mental flexibility). They conducted a factorial analysis of the Stroop test (which evaluates inhibition) and the Wisconsin Card Sorting Test (WCST) (which evaluates mental flexibility) and concluded that EFs are diversified, though interconnected. Based on this model, Diamond (2013) defined EFs as a specific set of abilities of attention regulation involved in conscious problem-solving led by objectives. Those abilities include cognitive flexibility, working memory and inhibitory control (Zelazo et al., 2016).

Inhibitory control involves thinking before acting, resisting temptations or habitual reactions, keeping focus and remaining reasonable, solving problems, being flexible in adjusting to demands or changing priorities, and seeing things from new perspectives (Diamond, 2013). Since they predict academic success, EFs have been the focus in studies of mindfulness programs for children (Diamond, 2010). Studies show evidence that training in mindfulness can enhance EFs in children (Meiklejohn et al., 2012; Tang et al., 2012; Schonert-Reichl et al., 2015). Among EFs, working memory, shifting and inhibition are some of the most studied among children, with dissociations reported in the age group studied here (Xu et al., 2013).

Mindfulness may be learned through practices such as mindfully paying attention to the breath to promote well-being, or learning to direct attention to the present experience and to learn to relate to it in a positive and purposeful way (Renshaw and Cook, 2017). Randomized controlled trials of MBI have associated improvements in EF components with activity in the anterior cingulate cortex and the autonomic nervous system (Tang et al., 2012).

In a study that implemented an 8-week mindfulness program (Paws b) with 71 7–9-year-old, Vickery and Dorjee (2015) reported significant improvement in metacognition and school performance, decreased negative feelings, and potential for the improvement of self-regulation and emotional well-being. In another study, the MindUP Mindfulness Program was tested in two classes and compared to another two classes that participated in a program focused on the promotion of social responsibility (BAU program). The children in the MindUP Program presented greater improvement in executive functions (EFs), self-report measures, well-being and prosocial behavior (Schonert-Reichl et al., 2015).

Other mindfulness-based curricular programs, namely, Mindful Schools (MS), Kindness (KC), Mindful Kids, and Mindfulness in Schools Program (MiSP), were also tested. The K5 curriculum of the Mindful Schools was applied in 409 elementary school children in public schools. Results showed increased attention, self-control, participation in activities and care and respect for others (Black and Fernando, 2014). The KC program, on the other hand, yielded improvements in social competence, in the domains of learning, health, and socioemotional development. The children in this study was 4.67 years old in average (Flook et al., 2015). The Mindful Kids program revealed some initial effects on stress and well-being immediately following training, and other effects became more visible at follow-up (Weijer-Bergsma et al., 2012). Finally, the MiSP yielded results in acceptability, depression symptoms, as well as reduced stress and improved well-being (Kuyken et al., 2013). Thus, mindfulness can lead to social and emotional gains when implemented as a practice in the school curriculum (Zoogman et al., 2014).

Reading and telling traditional tales to children can have significant benefits on children’s education and development, allowing them to identify with story characters and understand themselves and others (Hibbin, 2016). Reading and telling stories may address holistic perspectives, value emotional realities, link theory to practice, stimulate critical thinking skills and capture the complexities of situations by revealing multiple perspectives, encouraging self-evaluation and constructing new knowledge (Alterio, 2002).

Reading and telling stories has recently been resurfacing in education, contributing to strengthen social, educational, and affective bonds. While listening to stories, children may sympathize with the main character and use their imagination to solve problems (Shafieyan et al., 2017). Telling stories is effective in increasing happiness and resilience (Moghadam et al., 2016) and serves as a means to support children’s development (Soleymani et al., 2017). Consequently, it is important to invest in interventions that nourish children’s empathy with storybooks (Kucirkova, 2019).

While several meta-analyses reporting positive results have focused mainly on the use of MBI in schools, authors should not ignore other evidence-based interventions that may be positive in school settings. Research comparing the effectiveness of mindfulness with other interventions should be conducted to verify which is the best approach for improving academic skills and reducing disruptive behaviors in the classroom (Renshaw et al., 2017).

To the best of our knowledge, no previous studies have compared the effects of story reading intervention and MBI in a school setting. Although the science of MBI in schools is advancing, a research agenda should be developed involving implementation issues as well as the development of assessment-to-intervention practices grounded in biological and psychological processes, cultural factors, and the relation between MBI and other social-emotional competencies (Renshaw and Cook, 2017).

Thus, here we compared the effects of a mindfulness-based intervention (MBI) and story reading intervention (SI) on executive functions and emotions in a group of Brazilian public school students aged 8–9. We hypothesized that MBI may have a stronger effect on EFs due to its focus on attention and awareness and that SI may have a stronger effect on emotional health due to its focus on empathy and emotional conflict-solving. We also wanted to evaluate the feasibility of these interventions in Brazilian public schools.

## Methods

This was a controlled clinical trial without randomization, that compared two groups of children from two different public schools: a group of children who participated in a MBI (school 1) and a group who participated in a story reading intervention (SI) (school 2). Participants were matched by school grade and socioeconomic level. Randomization was not possible because children receiving one intervention should not be in contact with children receiving the other one.

### Participants

The population of eligible participants was 245 students. The inclusion criteria were being between 8 and 9 years old, being enrolled in one of the two schools, obtaining their family’s consent, and not having any mental disorder or special needs that interfered with their participation. The exclusion criteria were students who were away from school.

Thirty-eight students were excluded for the following reasons: three had autism, one had borderline personality disorder, one had Prader–Willi syndrome, one was not in the target age group, one took controlled medication and was very aggressive, one was in the middle of a family dispute (court case), and 10 had parents who did not provide consent. The final sample consisted of 207 students, 111 from school 1 (mean age = 8.93 ± 0.64) and 96 from school 2 (mean age = 8.98 ± 0.58). The two groups did not differ significantly from each other in terms of age, gender, religious belief, father’s or mother’s schooling or family income (*p* > 0.05) (Supplementary Material 1). The entire dataset is available as Supplementary Material 2.

### Procedure

This study conformed to the national and international norms of ethics for research with humans, according to resolution 466/12. It was conducted following approval by the Committee of Ethics in Research (CER) of the Universidade Federal de São Paulo (UNIFESP) under number 3.460.707. The identification of this study in the Registro Brasileiro de Ensaios Clínicos (Brazilian Register of Clinical Trials -REBEC) is RBR-8sdwws. After obtaining consent, we presented teachers, coordinators and assistants with an informative lecture on Mindfulness. There was no need to address Story reading, as all of them were familiar with it. In a second moment, teachers and family members participated in a meeting about the research instruments and the Mindfulness Intervention. Family members also answered a sociodemographic questionnaire.

The students and their parents signed a Consent Form agreeing to participate and the children were then instructed to answer the evaluation instruments. At baseline (T0), we applied the following instruments:

#### Sociodemographic Questionnaire

Sociodemographic questionnaire included questions about age, gender, religious beliefs, father’s or mother’s schooling, and family income.

#### CSS – Childhood Stress Scale

The Brazilian validation consists of 35 items on a Likert scale from 0 to 4 points. The items are grouped into four factors: physical reactions, psychological reactions, psychological reactions with depressive components and psychophysiological reactions. It comprises three factors of precision and internal consistency, with Alpha Cronbach’s coefficient of 0.89 (Lipp and Lucarelli, 2011).

#### CDI – Child Depression Inventory

Measures depressive symptoms via self-application in young people ages 7–17. In this version, the instrument consists of 20 items distributed among affective, cognitive, somatic, and conduct symptoms. Participants use a three-point Likert scale to indicate the best alternative describing their feelings during the previous 2 weeks. The Cronbach’s alpha of the Brazilian version is 0.91 (Coutinho et al., 2008).

#### MASC – Multidimensional Anxiety Scale for Children

Evaluates anxiety symptoms in children and adolescents and consists of 39 items that are answered on a Likert scale from 0 to 3. This questionnaire asks children what they have been thinking and doing and how they have been feeling recently, and tests four factors: physical symptoms, harm avoidance, social anxiety and separation anxiety. In the Brazilian version the internal consistency is α = 0.83 (March et al., 1997; Vianna, 2008).

#### CPANS – Positive and Negative Affect Schedule for Children

Composed of 34 items, with 17 items in each subscale. This scale is used to measure children’s emotions during the previous few weeks. The subscales measure positive and negative affect. The scale presents words that describe different feelings and emotions in a 5-item Likert scale ranging from 1 (“Very slightly or not at all”) to 5 (“Extremely”). The Alpha coefficients obtained for the Positive Affect (0.88) and Negative Affect (0.84) subscales attest to the reliability of the Brazilian version (Giacomoni and Hutz, 2006).

#### FDT – Five Digit Test

The FDT is an instrument used to assess an attention interference effect, called the Stroop effect, using conflicting information about numbers and quantities. The interference effect happens when two conflicting pieces of information about the same stimulus must be processed and the least automatic or intuitive option should be selected.

The FDT’s main measures are Reading, Counting, Choosing and Shifting. The first two are measures of automatic attention and processing speed and the last two are measures of controlled attention and executive attention. The Reading component is the simplest and presents digits in quantities that exactly match their values. The Counting component presents groups of one to five asterisks, and the individual must recognize the “set” and count the number of asterisks. In Choosing, the subject must inhibit reading the numbers presented and say how many numbers are present in each stimulus (there is an incongruency). Finally, in Shifting, one in five groups of digits is delimited by a thicker border. The individual is instructed to alternate between two operations, counting 80% of the items (as in Choice), but breaking this routine when the border is thicker. In addition to Reading, Counting, Choosing and Shifting, there are two executive indexes: Inhibition and Flexibility. The Brazilian version presented an internal consistency varying from 0.88 to 0.90 (Sedó et al., 2015). Better scores in FDT are reduced response times (less time to complete the task) and fewer errors.

The first instrument to be applied was the CSS, then the CDI, MASC, CPANS, and finally FDT.

Soon after FDT data collection at T0 (baseline), students in school 1 were submitted to the adapted curriculum K5 (MS) of the Mindful Schools (2014), which consists of 16 Mindfulness practices for 8 weeks (twice a week). At the same time, the students from school 2 had the Story reading Intervention – 16 stories in 8 weeks (twice a week). Immediately after the 8-week interventions (T1), we collected FDT data once again.

### Mindfulness-Based Intervention

The mindfulness-based intervention (MBI) in education was inspired by the K5 curriculum of the Mindful Schools (MS), with adaptations: we included some objects in the practice, such as a hula hoop, a gratefulness ball, an anchor for breathing, generosity buttons and rainsticks to facilitate students’ comprehension. A certified instructor guided the practices in the 16 meetings for 8 weeks. Seven of those meetings lasted 30 min (twice a week), and the last one lasted 60 min. Students were given diaries and were asked to write about the day’s session. They were also asked to practice the conscious abilities learned in their daily routine (i.e., in the classroom, at recess, on the bus and at home).

#### Mindfulness Classes

##### Week 1

Session 1 (Awareness): Mindfulness of one’s body and listening. Children were instructed to close their eyes, to be conscious of their own bodily sensations and the sounds in the environment.

Session 2 (Awareness): Mindful attention to breathing. Children were instructed to sit quietly, close their eyes and pay attention to their breathing.

##### Week 2

Session 3 (Heartfulness). Children learned about the importance of being generous and heartful and practiced sending loving thoughts to other people. They gave buttons to the person in their thoughts.

Session 4 (Awareness): Body awareness. Seated quietly, children were instructed to scan their whole body and observe feelings and sensations.

##### Week 3

Session 5 (Awareness): Mindfulness of the breath. Children were instructed to pay attention to their breathing and to use it as an anchor to deal with distressing situations. They received the breathing button to help them understand this ability as a resource they may use.

Session 6 (Heartfulness): Generosity. Children were instructed on how to be generous and at the end of the class received the heart button from their friends.

##### Week 4

Session 7 (Awareness): Thoughts. Children heard about how thoughts determine feelings and actions and about the importance of using our resources to deal with them.

Session 8 (Awareness): Observing with mindfulness. Looking around and finding things the eyes do not see.

##### Week 5

Session 9 (Heartfulness): Generosity: caring for others during recess. Visualizing good feelings toward other children during recess.

Session 10 (Awareness): Emotions making room. Identifying emotions and where they are felt in one’s body.

##### Week 6

Session 11 (Awareness): Slow motion movements. Awareness of movements doing slow motion movements.

Session 12 (Heartfulness): Gratitude practicing kindness. How to be grateful (with the gratitude ball) with feelings.

##### Week 7

Session 13 (Awareness): Mindful walking. Helping in the perception of sensations (with hula hoop)

Session 14 (Awareness): Mindful eating. Eating a raisin very slowly to feel the sensations it brings to the mouth as well its size and flavors.

##### Week 8

Session 15: Doing tests with mindfulness. Feelings before, during and after tests.

Session 16: Finishing. Mindfulness for life. Retrospective of the practices conducted with the students.

### Story Reading

Students from school 2 participated in the Story reading Intervention (SI). Sessions took place at the same time and on the same days as the Mindfulness Intervention for students in school 1. The stories were selected and told by a psychologist specialized in child development from the book “O Grande Livro das Emoções” by Pujol and Bisquerra (2012) and included the following themes: trusting oneself, recognizing emotions and feelings, appreciation, sensitivity, calming down, facing challenges, resilience, dealing with frustrations, preventing aggression, and kindness toward others. The psychologist told 16 stories for 8 weeks (twice a week) and each session lasted 15 min, emphasizing the moral at the end. However, there was no discussion with the children due to the short time for this activity (15 min).

#### Story Reading Classes

##### Week 1

Story 1: Little Chickpea (Grãozinho de Bico).

Moral: Trust yourself, be aware of limitations and fears.

Story 2: Pied Piper of Hamelin.

Moral: Recognize emotions and feelings.

##### Week 2

Story 3: My friend Blenka.

Moral: Instruction and education of sensitivity to be able to appreciate beauty.

Story 4: Dom Rodrigo’s dog.

Moral: We should not say “consider it done”; we should think before doing.

##### Week 3

Story 5: The two tailors.

Moral: Calming down is important to face life’s challenges.

Story 6: The painter Notxa was sad.

Moral: Understanding the sadness of others is a form of emotional control.

##### Week 4

Story 7: Aeneas and the destruction of Troy

Moral: Resilience has a lot to do with the attitude we have toward life.

Story 8: The three fishes.

Moral: It is important to increase tolerance to frustration.

##### Week 5

Story 9: John’s Leap (O pulo do JOÃO).

Moral: Good memories make life dynamic: “if I was able, if I was happy.”

Story 10: The bunch of grapes.

Moral: Those who have empathy do to others as they would have them do to themselves.

##### Week 6

Story 11: Cyrano de Bergerac.

Moral: It is necessary to beat shyness with will and determination.

Story 12: The revenge of Achilles.

Moral: The feeling of revenge should not generate violence.

##### Week 7

Story 13: Some do it one way, and some do it another.

Moral: We should be assertive to prevent aggressiveness.

Story 14: Haarlem, grateful city.

Moral: Being grateful is a duty.

##### Week 8

Story 15: Hatred and forgiveness.

Moral: It is important to learn to forgive. It makes us feel good.

Story 16: The window.

Moral: sharing emotions and memories, beauty and kindness.

### Statistical Analysis

Group differences were evaluated using chi-squared and Student’s *t*-tests, and an ANOVA for repeated measures was used to compare scores between and within groups at baseline (T0) and 8 weeks after the intervention (T1). All analyses were performed using the program JASP Team (2020, Version 0.14.1).

## Results

### Effects of the Interventions on Executive Functions (EFs)

Participants in both schools presented improved FDT scores from T0 to T1. However, a time × group effect revealed that school 1 (MBI) showed greater improvements on FDT scores for Reading (time) [*F*_(__1__)_ = 26.45, *p* < 0.001, η_*p*_^2^ = 0.114], Counting (time) [*F*_(__1__)_ = 41.21, *p* < 0.001, η_*p*_^2^ = 0.167], Choosing (time) [*F*_(__1__)_ = 71.27, *p* < 0.001, η_*p*_^2^ = 0.258], Choosing (errors) [*F*_(__1__)_ = 18.54, *p* < 0.001, η_*p*_^2^ = 0.083], Shifting (time) [*F*_(__1__)_ = 60.14, *p* < 0.001, η_*p*_^2^ = 0.227], Shifting (errors) [*F*_(__1__)_ = 15.96, *p* < 0.001, η_*p*_^2^ = 0.072], Inhibition Score [*F*_(__1__)_ = 20.16, *p* < 0.001, η_*p*_^2^ = 0.090], and Flexibility Score [*F*_(__1__)_ = 17.24, *p* < 0.001, η_*p*_^2^ = 0.078]. There was no difference on FDT scores for Reading (errors) [*F*_(__1__)_ = 0.16, *p* = 0.691, η_*p*_^2^ = 0.001], and Counting (errors) [*F*_(__1__)_ = 2.00, *p* = 0.159, η_*p*_^2^ = 0.010]. This information is presented in Table 1 and Supplementary Figures 1, 2.

**TABLE 1:** Comparisons between school 1 and school 2 for FDT scores in T0 (baseline) and after 8 weeks (T1).

	**School 1**		**School 2**		**Time effect**	**Group effect**	**Time × Group effect**	**Effect size (Time × Group)**	***Post hoc***			
	**(*n* = 111)**		**(*n* = 96)**						**comparisons**			
	**T0**	**T1**	**T0**	**T1**					**School 1**	**School 2**	**T0**	**T1**
				
**FDT scores**	**Mean**	**Mean**	**Mean**	**Mean**					**T0 × T1**	**T0 × T1**	**School 1 × School 2**	**School 1 × School 2**
	**(SD)**	**(SD)**	**(SD)**	**(SD)**								
Reading (time)	34.50 (9.70)	28.11 (6.56)	32.30 (8.18)	31.86 (7.30)	<0.001**	0.416	<0.001**	0.114	<0.001**	1.000	0.307	0.005**
Reading (errors)	0.09 (0.32)	0.00 (0.0)	0.08 (0.28)	0.01 (0.10)	<0.001**	0.932	0.619	0.001	0.015*	0.131	1.000	1.000
Counting (time)	45.69 (11.52)	34.92 (9.04)	41.72 (12.48)	38.56 (9.77)	<0.001**	0.904	<0.001**	0.167	<0.001**	0.002**	0.051	0.094
Counting (errors)	0.77 (1.60)	0.10 (0.43)	0.72 (1.05)	0.31 (0.83)	<0.001**	0.479	0.159	0.010	<0.001**	0.017*	1.000	0.921
Choosing (time)	71.44 (16.78)	51.13 (12.58)	65.66 (15.09)	59.42 (15.58)	<0.001**	0.516	<0.001**	0.258	<0.001**	<0.001**	0.038*	<0.001**
Choosing (errors)	3.46 (5.35)	0.23 (0.64)	2.16 (2.44)	1.40 (1.96)	<0.001**	0.844	<0.001**	0.083	<0.001**	0.433	0.020*	0.050
Shifting (time)	81.40 (20.35)	58.39 (14.31)	75.17 (16.32)	67.74 (16.99)	<0.001**	0.473	<0.001**	0.227	<0.001**	<0.001**	0.058	<0.001**
Shifting (errors)	3.74 (4.54)	0.44 (0.98)	2.98 (3.22)	1.91 (2.06)	<0.001**	0.267	<0.001**	0.072	<0.001**	0.055	0.435	0.003**
Inhibition Score	37.13 (15.72)	23.03 (10.35)	33.59 (12.76)	27.55 (12.50)	<0.001**	0.753	<0.001**	0.090	<0.001**	<0.001**	0.312	0.078
Flexibility Score	46.65 (19.46)	30.38 (12.15)	43.11 (14.00)	35.87 (14.47)	<0.001**	0.594	<0.001**	0.078	<0.001**	<0.001**	0.595	0.063

*p < 0.05; **p < 0.01; *n*, number of participants; T0, baseline; T1, after intervention at school 1; T2, after intervention at school 2; Size Effect, η_*p*_^2^ (Less than 0.01 indicates a small effect size, 0.06 indicates a medium effect size and greater than 0.14 indicates a large effect size) (Cohen, 1973); FDT, Five Digit Test.

### Effects of the Measures on Emotional Health

While there were no differences between schools in terms of emotional health at time T1 relative to baseline for MASC [*F*_(__1__)_ = 0.76, *p* = 0.385, η_*p*_^2^ = 0.004], CPANAS-P [*F*_(__1__)_ = 1.47, *p* = 0.226, η_*p*_^2^ = 0.007], and CSS [*F*_(__1__)_ = 0.77, *p* = 0.382, η_*p*_^2^ = 0.004]. However, there was a trend for reduced negative affect and depression as measured with the CPANAS-N [*F*_(__1__)_ = 3.69, *p* = 0.056, η_*p*_^2^ = 0.018],and CDI [*F*_(__1__)_ = 3.56, *p* = 0.061, η_*p*_^2^ = 0.017] for students in school 2 (Story reading). This information is presented in Table 2 and Supplementary Figure 3.

**TABLE 2:** Comparisons between school 1 and school 2 for MASC, CPANS, CDI, and CSS scores in T0 (baseline) and after 8 weeks (T1).

	**School 1**		**School 2**		**Time effect**	**Group effect**	**Time × Group effect**	**Effect size (Time × Group)**	***Post hoc***			
	**(*n* = 111)**		**(*n* = 96)**						**comparisons**			
	**T0**	**T1**	**T0**	**T1**					**School 1**	**School 2**	**T0**	**T1**
				
**FDT scores**	**Mean**	**Mean**	**Mean**	**Mean**					**T0 × T1**	**T0 × T1**	**School 1 × School 2**	**School 1 × School 2**
	**(SD)**	**(SD)**	**(SD)**	**(SD)**								
MASC	51.37 (15.57)	52.47 (15.59)	58.29 (18.26)	57.18 (18.25)	0.995	0.004**	0.385	0.004	1.000	1.000	0.021*	0.276
CPANS-N	35.87 (10.63)	35.53 (12.05)	38.92 (11.88)	35.48 (13.60)	0.020*	0.311	0.056	0.018	1.000	0.024*	0.424	1.000
CPANS-P	65.18 (11.66)	68.19 (9.90)	65.89 (13.71)	66.71 (12.19)	0.035*	0.780	0.226	0.007	0.090	1.000	1.000	1.000
CDI	7.91 (6.46)	8.38 (7.48)	10.07 (8.09)	8.65 (7.22)	0.341	0.172	0.061	0.017	1.000	0.322	0.207	1.000
CSS	31.54 (17.32)	32.82 (18.17)	41.99 (21.58)	40.62 (20.84)	0.977	<0.001**	0.382	0.004	1.000	1.000	<0.001**	0.025*

*p < 0.05; **p < 0.01; n, number of participants; T0, baseline; T1, after intervention at school 1; T2, after intervention at school 2; Size Effect, ηp^2^ (Less than 0.01 indicates a small effect size, 0.06 indicates a medium effect size and greater than 0.14 indicates a large effect size) (Cohen, 1973); MASC, Multidimensional Anxiety Scale for Children, CPNAS – N, Children’s Positive and Negative Affect Schedule – Negative Affects; CPNAS – P, Children’s Positive and Negative Affect Schedule – Positive Affects; CDI, Children’s Depression Inventory; CSS, Children’s Stress Scale.

## Discussion

The aim of this study was to compare the effects of a Mindfulness-Based Intervention (MBI) with Story reading Intervention (SI) on executive functions, anxiety, depression and stress symptoms, as well as positive and negative affect in 8–9-year-old public school students.

*Post hoc* analyses showed increased performance from T0 to T1 on all components of FDT for school 1 (MBI) and in Reading time, Choosing time, Shifting time and errors for school 2 (SI) (see Table 1). Among the cognitive skills showing improvement following MBI were automatic attention and processing speed (Reading and Counting) as well as controlled attention and executive attention (Choosing, Shifting, Flexibility and Inhibition). In line with these results, Schonert-Reichl et al. (2015) reported that the MindUP curriculum resulted in significant improvement in EFs in children compared with a social responsibility program. In our sample, MBI had significantly greater effects on EFs than Story reading.

Higher order EFs, such as reasoning, problem solving and planning (Collins and Koechlin, 2012; Janz et al., 2019) are developed and promote the exploration and creation of new behavioral strategies. They also help limit impulsive responses, regulate emotions, and prevent bad decisions (Blair, 2016). Previous studies have shown that practicing mindfulness can help support the development of EFs and self-regulation in childhood (Harnett and Dawe, 2012; Meiklejohn et al., 2012; Tang et al., 2012; Zelazo and Lyons, 2012; Schonert-Reichl et al., 2015), which is supported by the results presented in the current study.

According to Zenner et al. (2014), mindfulness practices might optimize education in the 21st century by shaping more committed citizens. It trains students’ self-awareness and relational abilities. According to Diamond (2010), when emotional, social and physical needs are met, EFs and the pre-frontal cortex are working.

Here we highlight the importance of mindfulness abilities for students to perform daily and school activities, helping them focus attention and helping them choose and inhibit information depending on the situation. As suggested by Tang et al. (2012), to improve specific EFs one must develop the capacity to think before acting and to eliminate impulsive behaviors in order to reach goals. This involves increasing awareness of thoughts, emotions and actions, and it may explain the differences we found in negative affect and stress.

The adapted version of the K5 (MS) curriculum, applied as the MBI in this study, addresses the constructs that have become the focus for many education professionals: attention and self-regulation. This program also deals with empathy, kindness, well-being, generosity, and compassion (Black and Fernando, 2014).

Concerning emotional health, this study evaluated anxiety and depression symptoms, positive and negative affect and stress. Interestingly, there were no differences between schools/interventions in terms of emotional effects at T0 or T1. However, there was a trend in the reduction of negative affect and depression symptoms at T1 in school 2 (SI) (Table 2).

Several studies have suggested that mindfulness training with children may promote functional alterations in the brain that allow better stress control and suggest such training should be included in the class curriculum (Zoogman et al., 2014; Felver et al., 2016; Tan, 2016; Bauer et al., 2019). While we did not observe significant contributions to emotional health in our study, a bigger sample in a randomized controlled study could unmask possible contributions of MBI and SI to emotional health.

Although we only observed a trend in this direction, the psychological themes of Story reading may have a positive effect on depression symptoms and negative affect. Story reading contributes to strengthen social, educational, and affective aspects of life (Shafieyan et al., 2017) and may increase happiness and resilience (Moghadam et al., 2016). Story reading helps listeners understand the essence of complex concepts and ideas. It has been used beyond the realm of public communication to add a deeper dimension to communication with students (Suzuki et al., 2018). In our SI, children listened to stories that ended with a moral statement about behaviors and feelings but were not stimulated to actively become aware of their thoughts and actions, as was done in our MBI. Thus, we expected that MBI should improve EF better than SI. Due to the moral contents about behavior and feelings, and the development of empathy toward the stories’ main characters, we expected some effects of SI on emotional health. Future studies should continue to compare the effects of mindfulness versus Story reading Interventions.

This study contributes to the growing literature on the implementation of new approaches in education, including mindfulness and story reading, to improve executive functions and emotional aspects in children ages 8–9 years old.

A limitation of our study was the impossibility to randomize the children, since they belonged to the same class, and it was not possible to split them to receive the interventions. We decided to choose different schools to avoid children sharing their experiences during the experimental process. Despite 245 students, we had only two schools involved in the analysis, and a larger number of schools could be involved in a next study. Scales evaluating more “positive emotions” would be applied in the sample.

In Brazil, few studies have been conducted on Mindfulness Interventions in 8–9-year-old students. The MBI applied in this study can be carried out in a relatively short time, not disrupting the students’ academic curriculum.

In conclusion, it is feasible to implement MBI or SI in Brazilian public schools. Mindfulness-Based Intervention may present broader effects on executive functions than Story reading Intervention. There were no differences between groups in depression, stress, positive and negative affect anxiety, but Story reading showed a trend in reducing negative affect and depression.

## Data Availability Statement

The original contributions presented in the study are included in the article/Supplementary Material, further inquiries can be directed to the corresponding author/s.

## Ethics Statement

The studies involving human participants were reviewed and approved by the Comitê de Ética Universidade Federal de São Paulo. Written informed consent to participate in this study was provided by the participants’ legal guardian/next of kin.

## Author Contributions

CM conceived and designed the study, collected the data, guided the interventions, collaborated in the analysis of the data, and wrote the article. EK made substantial contributions to the design of the study, collaborated in statistical analysis, and critically reviewed the manuscript for important intellectual content. SL contributed to the study design, facilitated data acquisition, and performed statistical analysis. CB and PT made substantial contributions to the study design and critically reviewed the manuscript for important intellectual content. AH guided the study in all stages, as well as critically reviewed the manuscript for important intellectual content. All authors read and approved the final manuscript.

## Conflict of Interest

CB and PT were employed by company ‘Natura Cosméticos, Cajamar, Brazil’. The authors declare that this study received funding from Natura Cosméticos S.A. The funder had the following involvement in the study: study design.

## Supplementary Material

Supplementary Figure 1 FDT [Reading (time), Counting (time), Choosing (time), Shifting (time) Inhibition, and Flexibility] scores obtained by school 1 and school 2 at baseline (T0) and after interventions (T1).

Supplementary Figure 2 FDT [Reading (errors), Counting (errors), Choosing (errors) and Shifting (errors)] scores obtained by school 1 and school 2 at baseline (T0) and after interventions (T1).

Supplementary Figure 3 Emotional health scores obtained by school 1 and school 2 at baseline (T0) and after interventions (T1).
