# Supplementary figures and images for: Mindfulness-Based Versus Story Reading Intervention in Public Elementary Schools: Effects on Executive Functions and Emotional Health
Source: Front Psychol. 2021 Jul 8;12:576311. doi: 10.3389/fpsyg.2021.576311 (PMC8299946; doi:10.3389/fpsyg.2021.576311)

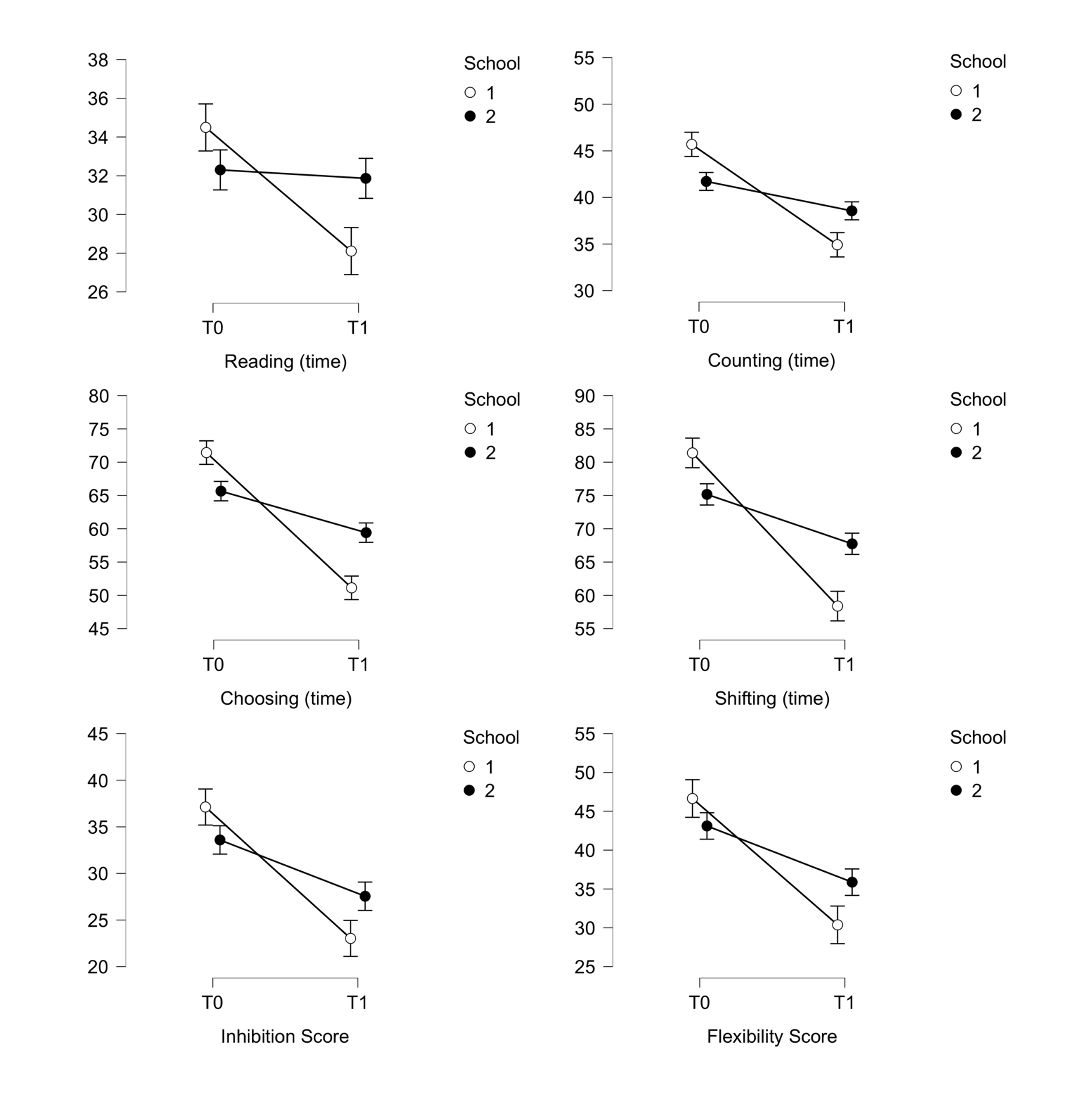

Supplement: Supplementary Figure 1 — FDT [Reading (time), Counting (time), Choosing (time), Shifting (time) Inhibition, and Flexibility] scores obtained by school 1 and school 2 at baseline (T0) and after interventions (T1). [file Image_1.TIFF]

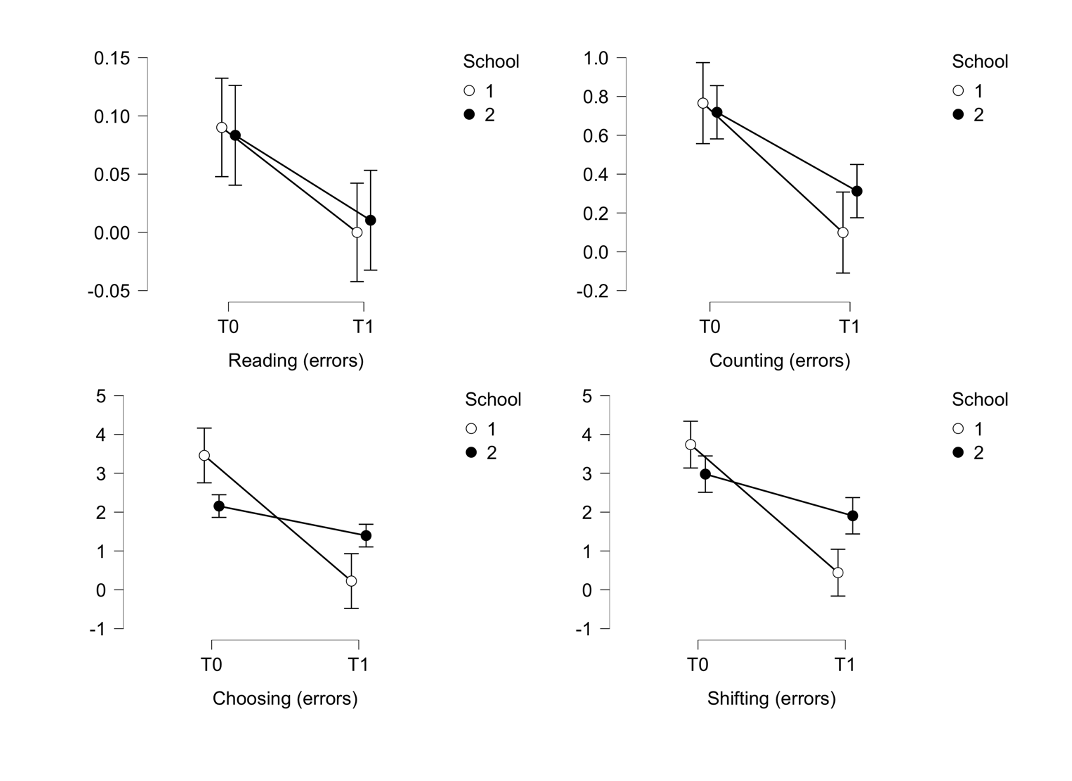

Supplement: Supplementary Figure 2 — FDT [Reading (errors), Counting (errors), Choosing (errors) and Shifting (errors)] scores obtained by school 1 and school 2 at baseline (T0) and after interventions (T1). [file Image_2.TIFF]

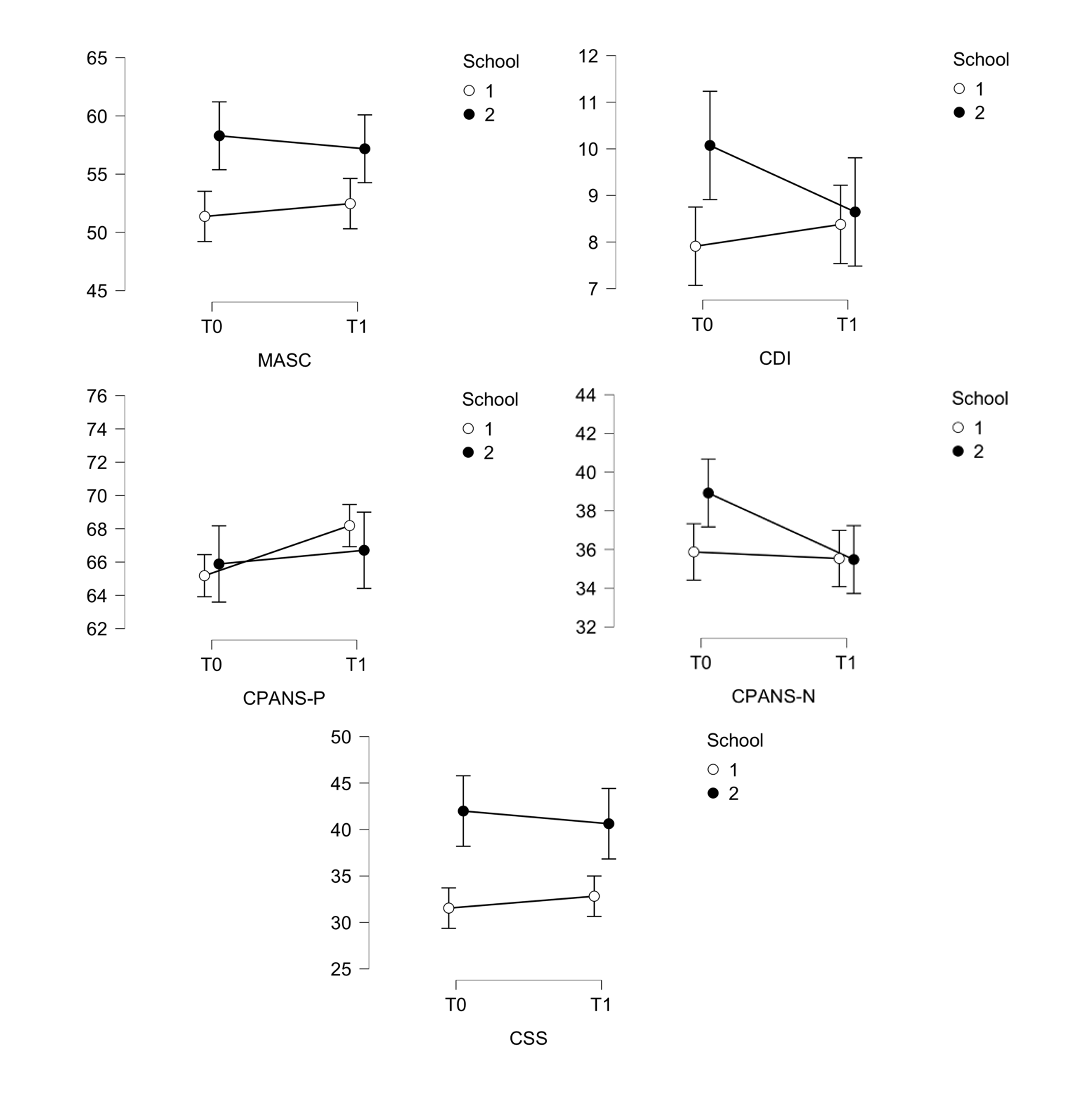

Supplement: Supplementary Figure 3 — Emotional health scores obtained by school 1 and school 2 at baseline (T0) and after interventions (T1). [file Image_3.TIFF]
