# Supplementary material for: Mindfulness-Based Versus Story Reading Intervention in Public Elementary Schools: Effects on Executive Functions and Emotional Health
Source: Front Psychol. 2021 Jul 8;12:576311. doi: 10.3389/fpsyg.2021.576311 (PMC8299946; doi:10.3389/fpsyg.2021.576311)
Supplement: Supplementary file 4 [file Table_1.DOCX]

**Supplementary material 1. Demographic characteristics of schools 1 and 2**

|  |  | **School 1 (n=111)** | **School 2 (n=96)** | **Sig.** |
| --- | --- | --- | --- | --- |
| Age | Mean (Sd) | 8.98 (0.58) | 8.93 (0.64) | 0.536^t^ |
| Gender | Male | 53.2% | 44.8% | 0.230^Q^ |
|  | Female | 46.8% | 55.2% |  |
| Religious belief | Catholic | 49.5% | 63.5% | 0.158^Q^ |
|  | Protestant | 27.0% | 14.6% |  |
|  | Spiritism | 5.4% | 4.2% |  |
|  | African Brazilian | 1.8% | 1.0% |  |
|  | Other religion | 5.4% | 2.1% |  |
|  | No religion | 10.8% | 14.6% |  |
| Father's schooling | Illiterate | 0.0% | 1.0% | 0.604^Q^ |
|  | Elementary School I | 11.7% | 16.7% |  |
|  | Elementary School II | 21.6% | 14.6% |  |
|  | High School | 43.2% | 45.8% |  |
|  | Graduate | 13.5% | 15.6% |  |
|  | Postgraduate | 3.6% | 2.1% |  |
|  | No Answer | 6.3% | 4.2% |  |
| Mother's schooling | Illiterate | 2.7% | 1.0% | 0.497^Q^ |
|  | Elementary School I | 4.5% | 8.3% |  |
|  | Elementary School II | 24.3% | 19.8% |  |
|  | High School | 49.5% | 51.0% |  |
|  | Graduate | 15.3% | 17.7% |  |
|  | Postgraduate | 2.7% | 0.0% |  |
|  | No Answer | 0.9% | 1.0% |  |
| Family Income | Less than 1 salary | 19.1% | 16.7% | 0.330^Q^ |
|  | 1 to 3 salaries | 52.7% | 52.1% |  |
|  | 3 to 6 salaries | 18.2% | 26.0% |  |
|  | 6 to 9 salaries | 3.6% | 4.2% |  |
|  | 9 to 12 salaries | 1.8% | 0.0% |  |
|  | 12 to 15 salaries | 0.9% | 1.0% |  |
|  | Greater than 15 salaries | 3.6% | 0.0% |  |

**Legend:** n= Number of participants**;** Sd= Standard deviation; ^t^=Student t test; ^Q^=Chi-squared test.
